# Patient-Centered Cancer Care Programs in Italy: Benchmarking Global Patient Education Initiatives

**DOI:** 10.1007/s13187-015-0805-4

**Published:** 2015-03-15

**Authors:** Ivana Truccolo, Chiara Cipolat Mis, Silvia Cervo, Luigino Dal Maso, Marilena Bongiovanni, Alessandra Bearz, Ivana Sartor, Paolo Baldo, Emanuela Ferrarin, Lucia Fratino, Maurizio Mascarin, Mario Roncadin, Maria Antonietta Annunziata, Barbara Muzzatti, Paolo De Paoli

**Affiliations:** 1 Scientific Directorate, CRO Aviano National Cancer Institute, via F. Gallini 2, 33081 Aviano, PN Italy; 2 CRO-Biobank, CRO Aviano National Cancer Institute, via F. Gallini 2, 33081 Aviano, PN Italy; 3 Clinical Cancer Pathology, CRO Aviano National Cancer Institute, via F. Gallini 2, 33081 Aviano, PN Italy; 4 Epidemiology and Biostatistics, CRO Aviano National Cancer Institute, via F. Gallini 2, 33081 Aviano, PN Italy; 5 Associazione Nazionale Guariti Lungoviventi Oncologici (ANGOLO), CRO Aviano National Cancer Institute, via F. Gallini 2, 33081 Aviano, PN Italy; 6 Medical Oncology, CRO Aviano National Cancer Institute, via F. Gallini 2, 33081 Aviano, PN Italy; 7 Pharmacy, CRO Aviano National Cancer Institute, via F. Gallini 2, 33081 Aviano, PN Italy; 8 Radiotherapy, CRO Aviano National Cancer Institute, via F. Gallini 2, 33081 Aviano, PN Italy; 9 Psychooncology, CRO Aviano National Cancer Institute, via F. Gallini 2, 33081 Aviano, PN Italy

**Keywords:** Patient education, Neoplasms, Patient participation, Patient-centered care, Empowerment, Comprehensive cancer centers, Ethical issues: cancer education

## Abstract

In Italy, educational programs for cancer patients are currently provided by the national government, scientific societies, and patient advocate organizations. Several gaps limit their effectiveness, including the lack of coordinated efforts, poor involvement of patient feedback in the planning of programs, as well as a lack of resources on innovative cancer-related topics. This process is parallel to a strong shift in the attitude of patients towards health in general and taking charge of their own health conditions in particular. The National Cancer Institute in the USA and the Organization of European Cancer Institutes encourage comprehensive cancer centers in providing educational programs conceived to overcome these gaps. The goal of this paper is to identify and describe the key elements necessary to develop a global patient education program and provide recommendations for strategies with practical examples for implementation in the daily activities of cancer institutes. A multidisciplinary committee was established for patient education, including patient representatives as equal partners, to define, implement, verify, and evaluate the fundamental steps for establishing a comprehensive education program. Six essential topics were identified for the program: appropriate communication of cancer epidemiology, clinical trial information, new therapeutic technologies, support in the use of medicines, psycho-oncological interventions, age-personalized approaches, and training programs for healthcare providers. Integration of these topics along with patient feedback is the key to a successful model for educational programs. An integrated educational program can transform a comprehensive cancer center to an institution that provides research and care for and with patients.

## Introduction

Effective patient education requires an approach with a combined effort between healthcare providers and patients in preparing educational resources and programs. This concept is embedded in the current implementation of patient-centered care, an approach to care focused on individuals which are part of the extensive model of personalized medicine to better diagnose and cure patients [1, 2]. After a review of the current literature regarding the various patient education models, evidence shows a shift from a unidirectional medical-centered model to a bidirectional patient-centered one, where the proactive role of patients is considered more and more essential [3, 4], with involvement from various healthcare providers.

The importance of patient education is particularly relevant in the field of oncology care, where medical information is very complex, the individualized care is even more complex. Care decision may have significant and long-term implications for patients’ health [5] as well as their family caregivers. Currently, patient education resources and programs are developed by governmental agencies, scientific societies, and patient advocates in several countries [6, 7]. Despite this growing trend, some relevant aspects of oncology are not adequately covered by these programs, such as the appropriate communication of the epidemiology of cancer and their implications and of basic and translational research results in promoting diagnosis and treatment, the rationale for developing new drugs and their application in innovative therapeutic protocols, the possible role of integrated authoritative sources, both physical and virtual, to provide patients with reliable information [8, 9], along with the best ways to promote effective cancer patient involvement [10].

Many patient education programs show additional concerns, including a lack of integrated and coordinated approach, the paternalistic approach to educating patients (according to a “top-down” approach), and a lack of specific training designed for healthcare personnel. Moreover, there is a lack of data from patient feedback and/or patient survey of needs to assist in designing educational activities [6, 7]. These and other deficiencies were considered for the development of a recommended model of a global patient education program.

For the development of the global patient education program, the concept of P4 medicine—predictive, personalized, preventive, and participatory—recently changed to “P5 medicine,” with the fifth P standing for the psycho-cognitive aspects [11], is crucial. The primary objective is to maximize wellness for each individual in every condition rather than simply to treat their disease [12]. Presently, it is not clear how patients and members of the healthcare community can be aligned to collaborate to achieve the goals of P4/P5 medicine. The National Cancer Institute in the USA and the Organization of European Cancer Institutes (OECI) both acknowledged the necessity for medical institutes to be equipped with infrastructures and programs specifically aimed at the improvement of patient education [6, 7, 13, 14]. To overcome the lack of strategy and a model, OECI encourages Comprehensive Cancer Centers (CCCs) to develop programs to reinforce and integrate patient education activities [12] (see the OECI Accreditation Program, Chapter 6). CCCs include both hospitals and academic institutions with a high level of expertise in clinical, laboratory, and population-based research. One of their goals is to establish regular collaboration with other centers in order to exchange information, modify discipline-specific approaches, and integrate various specialties to achieve common scientific and clinical goals so they may represent an appropriate setting for developing patient education models.

To establish such a program, a prototypical Patient Education Committee (PEC) was appointed in 2010 at the National Cancer Institute CRO Aviano, one of the ten Italian CCCs. This PEC is a multi-professional [15], not hierarchical, team, including medical oncologists, research nurses, psycho-oncologists, pharmacists, biologists, librarians, patients, and volunteers. Patient representatives are key components for the entire patient education process: involved in planning, implementing, testing, and evaluating the various educational activities. Since the elements that constitute a patient-centered educational program may be heterogeneous, the PEC identified six major topics to be covered that are strongly related each other and to the translational research mission of the CCCs. This paper describes all these topics on a point by point basis and gives some key recommendations regarding program implementation in the CCCs and other healthcare facilities. The global aim is to improve the educational competence of patients and to provide professionals with specific strategies for communicating in a way that motivates patients to take action [16] and strives to reduce the distance between doctors and patients [17] as well [18, 19].

## Method

### Planning the Global Patient-Centered Education Program

Considering that patient education is a complex concept, we subdivided the development of the program into simple steps based on the following key elements:

1. *Health literacy*: The degree to which individuals have the capacity to obtain, process, and understand basic health information and services needed to make appropriate health decisions (Mesh, i.e., PubMed Medical Subject Headings’ definition). To fully understand the degree of health literacy in patients and their need of further information on health matters, it is essential to carry out investigations addressed to different population targets [20, 21].
2. *Empowerment*: A process that helps patients gain control over their lives, increasing their capacity to act on issues that they themselves define as important; a process through which patients *individually and collectively* are able to express their needs, present their concerns, devise strategies for involvement in decision-making, and take action to meet those needs [22].
3. *Information*:
  - *Information access modality*: still is too often taken for granted. “Individual’s rights to obtain and use information collected or generated by others” (Mesh)
  - *Information services*: organized services based on supplementary information an individual might have through databases, human resources, and other (i.e., Web 2.0 or subsequent) technologies.

The special attention to “information access” is a milestone of this process. Since the concepts of information and communication are frequently confused, it is fundamental to keep in mind their differences and similarities: information is the content, communication is the process in which the informative content is included in a circular process as part of an individual relationship. In the Italian CCCs, information is currently delivered through Patients Libraries and Patients Info Points [10]. While the libraries are managed by Patients Librarians, whose task is to receive information requests, interpret them and provide tailor-made responses, the Info Points are facilities managed in collaboration with associations of patients and of volunteers where preformed educational material is available. This organization may contribute to reduce the barriers on information, diagnosis, treatment, and rehabilitation services, thus favoring patient empowerment [21].

4. *Availability of patient education handouts and popular works*: i.e., leaflets, cds, booklets, pdfs, videos, etc. (see next paragraph).
  - Patient education handout: *Works consisting of a handout or self-contained informative material used to explain a procedure or a condition or the contents of a specific article in a biomedical journal and written in non-technical language for the patient or consumer*.
  - Popular works: *Works written for non-professional or lay audiences* (Mesh definitions).
5. *Educational models*: or rather the study of different “therapeutic education” modalities. “Theoretical models which propose methods of learning or teaching as a basis or adjunct to changes in attitude or behavior. These educational interventions are usually applied in the fields of health and patient education but are not restricted to patient care” (Mesh).

After careful review of the literature and discussion, we have identified six topics that should be part of an institutional program, either because they are specifically part of the mission of CCCs or because they are not exhaustively covered by currently available programs [6, 7].

Selected topics include the following: (a) cancer epidemiology for patients, (b) clinical trials, (c) medicine use review and the role of the oncology specialty pharmacist, (d) age-personalized communication and medicine, (e) psycho-oncology, and (f) teaching strategies for healthcare providers. The following sections are dedicated to explaining the importance of introducing each topic in the institutional program of CCCs and to outline the guidelines for implementing into practice.

### Educational Material Preparation Recommendations

**Fig. 1:**
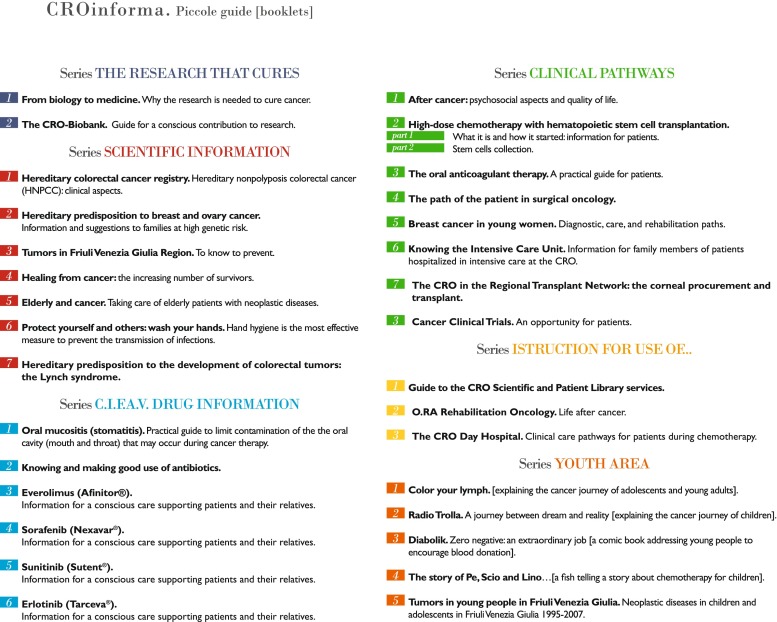
Overview of “CROinforma” booklets of popular science information, in Italian, published by CRO Aviano; their targets are patients and citizens, and their topics relate to research, prevention, and treatment of cancer (size: about 50–80 pages, with figures and schemes, A5 format)

The European School of Oncology (ESO), in their “Cancer on the Internet” conferences, recommended cancer organizations to provide patients with reliable, understandable, and accessible informational materials about cancer. To implement the plan at CRO Aviano, we prepared educational materials for topics, including booklets (Fig. 1), posters, videos, handouts, and internet resources, paying special attention in making them easily readable to users. To achieve this goal, planning occurred through a systematic coordinated action including the following:

1. An evaluation of the readability and communication style of the educational material, conducted by a multi-profession team including patients.
2. A strong network of reference information points and other dissemination tools and strategies to ensure the accessibility of the educational material in the CCCs and other locations.
3. An efficient feedback system is necessary. A successful example of an efficient way to get patient feedback has been recently implemented by the Radiotherapy unit of the CRO Aviano: a touch screen monitor has been placed in the entrance hall, so that patients can express their satisfaction/dissatisfaction opinion by simply touching a display. Using this method, patients are encouraged to answer. This method provides a method of capturing raw statistical data to use for improving the educational services.

#### a) Topic: Cancer Epidemiology for Patients

##### Rationale

An epidemiological education (i.e., on the frequency, causes, and cancer risk factors) can make patients aware of their choices and possibilities for preserving their health and their quality of life or often to reduce anxiety about cancer as a fatal disease.

##### Recommended Program

The patient education plan on the topic of epidemiology focuses on two main points: disease management and cancer prevention.

The first task is then to plainly describe the clinical statistical data about the disease (including neoplasia frequency, incidence,^1^ and prevalence,^2^ probability of patient survival and mortality data) to create awareness and avoid triggering unjustified fears or feeding unrealistic hopes. This includes providing patients with reliable data on the outcomes and implications of different treatment options, in order to help them make informed choices considering both the expectations of survival and their quality of life.

The second task of epidemiology in the patient education program is to effectively describe the evidence-based causes of cancer. Explaining the risks due to exposures or behaviors that contribute to cancer development or recurrence is at the root of educating patients towards modifying lifestyle habits.

The “Epidemiology for Patients” plan implemented by the PEC is delivered through a communication strategy based on booklets “Healing from cancer” and “Tumors in Friuli Venezia Giulia Region: Know how to prevent” (Fig. 1), leaflets, and educational sessions. At CRO Aviano, educational sessions were performed through face-to-face meetings between doctors and patients in the waiting rooms of the day hospital and clinical divisions. Patients waiting for their visit or treatment and during hospitalization have the option to participate in a session about a specific cancer type or related topics and to ask questions of the specialist. These sessions have proved to be a great success according to the number of patients and others attending and participating. Evaluation of the program and feedback including their requests for further topics to be covered are currently being developed for future implementation.

#### b) Topic: Clinical Trials

##### Rationale

Educational tools and information about clinical trials and other new clinical experimental approaches are inconsistently available or poorly understood by patients, particularly in oncology [23]. In order to respect the conscious choice principle, a patient agreeing to participate in a trial must be aware of the possibility to participate, the side effects of anticancer therapies, the possibility of being assigned to the control group, the right to withdraw, the characteristics of clinical trial phases, and the role and importance of patient representatives in ethical committees. Information about new technologies for research and treatment can be even more difficult to understand for patients, and so requires special attention.

##### Recommended Program

The first step to establish a clinical trial educational program in a CCC is to conduct a needs assessment of patient’s current knowledge, actual needs, fears, and expectations. Given their expertise in translational research programs, CCCs should provide information on specific items related to their mission, rather than duplicate information on the above-mentioned topics, and particularly provide detailed information on trials and instruments adopted in their own center. We suggest including information on the role of research in cancer treatment, state of the art of patient-centered cancer research, new drug discoveries, the role of pharmacogenomics, and the impact of new technologies [24].

One example of such new instruments established at CRO Aviano is the CRO-Biobank, an organizational structure aimed at collecting human biological samples for cancer (or related fields) research purposes. To contribute to a well-informed decision-making process and to improve patient involvement, CRO-Biobank implemented a novel multi-source informed consent procedure for patient enrolment, investigating participant needs and background, and the effects of multiple approaches. This information system reported outcomes with high rates of understanding (>99.0 %) and also of awareness (95.5 %) of study participation, even among less educated people [25].

For other projects, we prepared educational materials, including booklets (Fig. 1), posters, videos, CDs, and internet resources and trained the staff on specific trials to better answer patient questions and trigger further requests. Finally, to promote our mission in cancer research, we created “From biology to medicine: Why the research is needed to cure cancer” (Fig. 1), a booklet including the most recent progress made in our bench-to-bedside programs, innovative research technologies, and some “omics topics,” which are generally not easily understood by patients.

#### c) Topic: Medicine Use Review and the Role of Oncology Pharmacists

##### Rationale

For a CCC, the ability to provide independent, transparent, and high-quality information on medicines is essential, especially considering the various types of innovative antitumoral therapies available today. Hospital specialty pharmacists have a complete picture of the patient’s therapeutic course and may help them to thoroughly understand and manage their therapy.

##### Recommended Program

Hospital specialty pharmacists should be involved in a continuous process of patient education directly involving patients through the “Medicine Use Review” (MUR), a structured interview with the aim to assess and increase knowledge, agreement, and compliance to therapy [26], by checking prescriptions, drug interactions, and possible side effects. During the MUR interview, patients can express concerns and obtain information on the efficacy and safety of the medicines they were prescribed.

Furthermore, brochures, booklets (Fig. 1), and informative materials can serve as additional support to deliver appropriate general information on cancer therapies and prevention, written according to the needs and preferences expressed by patients in the clinical setting or in the discussion groups. At CRO Aviano, a specialty pharmacist is involved part time in the Information Point of the Patient Library in order to welcome patients and answer specific questions about drugs. To increase the patient reporting and understanding of adverse effects of their cancer therapies is a secondary aim of this program as well.

#### d) Topic: Age-Personalized Approach: Older Adults and Adolescents and Young Adults (AYA)

At any age, cancer patients ask for a more active participation in their treatment course and show an increasing need for information that should be met by healthcare workers [20, 27]. Yet, patients of different age groups have different training needs and require different approaches, especially older adults and adolescents and young adults (AYAs).

Older Adult Patients:

##### Rationale

More than 65 % of all cancers involve adults aged 65 and over; thus, it is critical to focus on this subgroup which represents an ever-increasing part of cancer patients. Older adult cancer patients are the population at higher risk for poor communication with health professionals, since they have lowest health literacy rates among different patient populations [28]. The absence of a specific education program for elderly patients may deeply impact diagnostic and therapeutic efficacy.

##### Recommended Program

We propose to set up close collaboration with cancer patient associations since they are essential to “taking care of those who care,” especially to support caregivers who often do not have a proper understanding of the real needs for information of their patients [29].

Adolescents and Young Adults (AYAs):

##### Rationale

“Emerging adulthood” is a phase of life where AYAs develop their own social identity, establish autonomy from parents, form strong peer relationships, and create new intimate and sexual interactions. Therefore, it requires a specific professional healthcare approach [30]. Taking into account the clinical and psychosocial characteristics of this age group, developing an educational program based on adequate information modalities for teenagers could help AYA patients feel at the center of their healthcare program and encourage adhesion to treatments. We believe this is fundamental in helping young patients live well despite their illness and return to a positive life routine as their treatment is completed.

##### Recommended Program

In treating adolescents with cancer, a comprehensive approach is necessary to take charge of all patient and caregiver needs. The healthcare team should work with the patient, considering him/her as a developing social entity. A specific program was established for this patient population named “Youth Area Project,” which was activated in January 2007 at CRO Aviano [31]. Success requires an improved cooperation between pediatric and adult oncology departments, the development of AYA specific postgraduate continuing medical education programs, a dedicated AYA hospital area, a physical age-appropriate environment, psychosocial support services, and the use of technology to educate patients and improve communication between patients and healthcare professionals. The sense and value of normalcy for the AYA patient are among the highlights of this dedicated program. Prior to opening the new ward, patients were involved in the choice of facilities and colors for this particular unit (“architecture humanization”). Educational support was promoted by interactive communication through typical age-group communication tools including e-mails, short message services, blogs, social networks (e.g., Facebook), diaries, and peer support groups [32]. This was in an effort to help sharing experiences and emotions among AYA patients and the activation of a “school-in-hospital” with home education services. We also developed an awareness campaign on the “Youth Area Project” with peer-to-peer conferences.

Finally, by encouraging and actively involving the AYA patients in their treatment, including the results of their laboratory testing or examinations for evaluating alternative treatments when required, a “dynamic culture” of informed consent was developed. These patient education activities allowed patients to process information on their personal experience [23], disseminate information on cancer in young adults, and give patients the chance to meet other AYA cancer survivors.

Topic: Psycho-oncology:

##### Rationale

Psycho-oncology has a crucial role in the personalization of cancer treatment for its specific mission: to promote self-awareness of the body/mind unit, to legitimize the expression and communication of emotions and experiences (apart from the expression of physical concerns and the organs affected by the disease), and to make patients responsible for taking an active role in caring for all aspects of their person (bio-psychosocial spheres) during and after the disease.

##### Recommended Program

Reaching the educational goals require the following: integration of psycho-oncology in clinical activities recommended by international guidelines, research and educational activities for understanding less investigated fields such as “life after cancer” issues, and training of healthcare staff.

As part of a multi-center research program funded by the Ministry of Health, the first Cancer Survivor Clinic (CSC) was founded at CRO Aviano. The CSC is specifically dedicated to long-term survivorship focused on the multidisciplinary assessment of patients who have been disease-free for at least 5 years. The “natural” development of this program is the codification/implementation of a multidisciplinary clinical operative model that may also become a model for the education/training of health professionals and for patients who are experiencing long-term survivorship. As a result of the program, psychometric tools were used to assess the primary psychosocial dimensions and quality of life [33] including the identification of patients at higher psychological risk. An informational booklet “After Cancer” (Fig. 1) was created which includes information on all the key cancer-related physical, psychological, social, and spiritual concerns that patients may be experiencing. Strategies and resources for the management of these primary concerns were included in the program.

#### e) Topic: Teaching Strategies for Healthcare Providers

##### Rationale

There is evidence that good communication between healthcare providers and patients is essential for high-quality treatment and that effective communication positively affects adherence to treatment, pain management, and psychological functioning [34]. This program topic is essential and overarching to all other topics. Healthcare provider training should not disregard the patient education aspect, which is frequently not included in doctors, nurses, and other healthcare providers’ curricula. Even though not all providers possess excellent relational capabilities, effective communication skills can be taught and remain unaltered over time [27].

##### Recommend Program

CCCs should promote experimental paths in patient education, meant to educate healthcare providers on specific aspects, including understanding of the importance of actively involving patients in their treatment and knowledge of the available resources (with their limitations) for patients. This may include the following: computer resources, brochures, websites, forums, families, media, and social assistance resources provided by government programs; promotion of preventive medicine and of positive lifestyle choices and early recognition of disease onset; and acquisition of an efficient ability to educate patients on cancer treatments (including symptom management) to reduce requests and to increase patient empowerment [17].

Educational training is recommended for social workers, nurses, doctors, psychologists, and information/communication experts. The National Cancer Institute (NCI) in the USA has a well-established training program structured as a 3-day intensive workshop, with the explicit goal of “educating the educators” in developing educational activities in their own care facilities [35]. In turn, participants are encouraged to involve other professionals from their own institutions and share the content and techniques learned.

Recommendation is to have more than one healthcare provider take part in training his/her peers to make the experience productive. The training model should be based on the principles of adult education including the use of audiovisuals to simulate interactions with patients, simulation and role-playing situations followed by other participant feedback, as well as experiences with real patients. The transmission of the contents should include teaching the basic principles of human communication, learning active listening techniques, and acquiring knowledge of the resources available to patients and families [36].

Monitoring the effectiveness of education and the retention of learned skills is necessary and possible through interviews and questionnaires with healthcare personnel and patients. Evaluation should be measured before the training course and again some months later. Achieving and maintaining competence learning, patient satisfaction in their relationship with healthcare personnel, better adherence to treatment, and an improved ability of patients to express their own informational needs, preferences, and feelings are positive indicators for the effectiveness of the patient education training program for healthcare providers.

For CCC staff to actively participate in patient education efforts, it is necessary to evaluate the sustainability and limits. Patient education programs and the role of the healthcare provider participation should be formally recognized by the CCC leadership and be integrated into everyday workflow, while avoiding any overload on the staff: “do it differently, not more*.*”

## Conclusions

Planning, implementing, evaluating, and acting a patient education program requires the effort of many professionals as well as others. The successful patient education program model includes an extension of the idea of personalized treatment using a concept analogous to P5 medicine [11]; it also requires the involvement of patients from the early stages of program design and multi-professional and interdisciplinary planning. Among the many topics of patient education in oncology, we identified six key issues that take into consideration clinical and translational CCCs research activities and roles. These issues could become an integral part of a user manual containing useful information for establishing an educational program in oncological institutions. CCCs should be pilots in establishing patient education programs involving the entire organization.

In order to insure constant improvement of educational programs and of their ongoing assessment, future developments should consider the creation of focus groups and/or educational sessions and/or classes with patients, systematic feedback from patients and others about the different activities and their utilization so that the activities can be improved. Further implementation goals should involve stronger networking with patient advocacy groups and scientific societies, to avoid overlapping programs and instead encourage collaborative ones and mutual exchanges. In addition, we believe that collaboration between the patient education program and the Office of Continuing Medical Education is required in order to develop educational programs for healthcare providers that take into account patient informational needs, as well as their preferences and priorities.

In conclusion, special attention must be paid to benchmarking. International benchmarking within and outside of Italy regarding the educational activities for patients at CCCs may provide relevant inputs to improving the quality and efficiency similar programs. Wherever this patient education model project has been planned and experimented with, adherence by healthcare providers and patients was reported to be remarkably good. For this reason, it is extremely important to continue on planning future activities, implementing the project and informational offers and services for patients. At the same time, it is essential to create a network of needs assessment surveys to assess stakeholder satisfaction. Therefore, based on initial evaluations, we would like to concentrate our efforts on establishing the most appropriate tools to personalize the approach to the needs of cancer patients. In our view, the rate of success in daily delivery of healthcare could well be determined by patient access to the best diagnostic and therapeutic tools and also by the way institutions respond to a growing demand for a more active role by patients in managing their disease, with the end goal of better quality of life.

## Footnotes

1 The possible number of people who will contract a disease in a given area.

2 The number of people that will live with a tumor.
